# Differential gene expression patterns during gametophyte development provide insights into sex differentiation in the dioicous kelp *Saccharina japonica*

**DOI:** 10.1186/s12870-021-03117-z

**Published:** 2021-07-14

**Authors:** Jiaxun Zhang, Yan Li, Shiju Luo, Min Cao, Linan Zhang, Xiaojie Li

**Affiliations:** 1 School of Marine Science and Engineering, Qingdao Agricultural University, Qingdao, 266109 China; 2 National Engineering Science Research & Development Center of Algae and Sea Cucumbers of China, Provincial Key Laboratory of Genetic Improvement & Efficient Culture of Marine Algae of Shandong, Shandong Oriental Ocean Sci-Tech Co., Ltd., Yantai, 264003 China

**Keywords:** Sex differentiation, Dioicy, Brown algae, *Saccharina japonica*, Sex-biased gene, Sex-specific gene, Sexual dimorphism

## Abstract

**Background:**

In brown algae, dioicy is the prevalent sexual system, and phenotypic differences between male and female gametophytes have been found in many dioicous species. *Saccharina japonica* show remarkable sexual dimorphism in gametophytes before gametogenesis. A higher level of phenotypic differentiation was also found in female and male gametes after gametogenesis. However, the patterns of differential gene expression throughout gametophyte development and how these changes might relate to sex-specific fitness at the gamete stage in *S. japonica* are not well known.

**Results:**

In this study, differences in gene expression between male and female gametophytes in different developmental stages were investigated using comparative transcriptome analysis. Among the 20,151 genes expressed in the haploid gametophyte generation, 37.53% were sex-biased. The abundance of sex-biased genes in mature gametophytes was much higher than that in immature gametophytes, and more male-biased than female-biased genes were observed in the mature stage. The predicted functions of most sex-biased genes were closely related to the sex-specific characteristics of gametes, including cell wall biosynthesis, sperm motility, and sperm and egg recognition. In addition, 51 genes were specifically expressed in males in both stages, showing great potential as candidate male sex-determining region (SDR) genes.

**Conclusions:**

This study describes a thorough investigation into differential gene expression between male and female gametophytes in the dioicous kelp *S. japoni*ca*.* A large number of sex-biased genes in mature gametophytes may be associated with the divergence of phenotypic traits and physiological functions between female gametes (eggs) and male gametes (sperm) during sexual differentiation. These genes may mainly come from new sex-biased genes that have recently evolved in the *S. japonica* lineage. The duplication of sex-biased genes was detected, which may increase the number of sex-biased genes after gametogenesis in *S. japonica* to some extent. The excess of male-biased genes over female-biased genes in the mature stage may reflect the different levels of sexual selection across sexes. This study deepens our understanding of the regulation of sex development and differentiation in the dioicous kelp *S. japonica*.

**Supplementary Information:**

The online version contains supplementary material available at 10.1186/s12870-021-03117-z.

## Background

Brown algae, photoautotrophic and multicellular marine organisms, belong to the group Stramenopiles, which has been evolving independently of the well-studied animal, fungal and green plant lineages for over a billion years [1, 2]. Due to their unique evolutionary history, brown algae exhibit some remarkable characteristics. Compared with the fact that only 6% of flowering plants are dioecious [3, 4], separate male/female sexes are prevalent in brown algae, which may be partly related to the lack of a self-incompatibility system in brown algae [5]. In addition, remarkable diversity in the levels of sexual dimorphism between males and females, including isogamous, anisogamous and oogamous species, were exhibited in brown algae [5]. Accordingly, there has been a growing interest in sex chromosome evolution and the regulation of sex determination in brown algae in recent years [1].

Dioecy and dioicy are the two common types of brown algal sexual systems, in which separate sexes occur during the diploid and haploid phases of the life cycle, respectively. Of them, dioicy is prevalent in brown algae, especially in the Ectocarpales and Laminariales orders [1, 5]. In comparison with dioecious species with diploid-phase sex determination systems (XX/XY or ZW/ZZ system), the sex of these dioicous species is determined by a UV sex determination system, in which the U and V sex chromosomes are present in female and male gametophytes, respectively [6, 7]. Recent studies have been conducted on the origin and evolution of UV chromosomes in the brown algae and on the regulation of sex determination and sexual dimorphism in *Ectocarpus*, a model brown alga belonging to Ectocarpales [2, 8–11]. The sex-determining region (SDR) of both the female (U) and the male (V) sex chromosomes in *Ectocarpus* has been identified and characterized [2]. The male and female SDRs evolved independently by more than 70 million years and are highly diverged at the sequence level. However, similar sizes and structures were observed between U and V SDRs [1, 2]. In addition to the low gene density, a large amount of repeated DNA in the SDR and the longer introns in SDR genes, they are of a similar estimated size (0.93 Mb and 0.92 Mb for female and male SDR, respectively) and are relatively small, constituting only one-fifth of the sex chromosome. In addition, only a small proportion of sex-biased genes (< 12%) were detected during gametophyte generation, and similar evolutionary rates were shown for male- and female-biased genes [8, 12]. All of the above similar features shared by both U and V SDRs were consistent with the low level of sexual dimorphism of *Ectocarpus*.

*Saccharina japonica* is one of the most commercially important brown algae that has been cultivated in China. Its annual farming production has reached over 1 million tons, providing raw materials for the food and pharmaceutical industries [13, 14]. In addition, cultivated *S. japonica* has brought considerable environmental benefits by removing nitrogen and phosphate, sequestrating carbon and releasing O_2_ [15]. *S. japonica* belongs to the Laminariales order, and the sexes of its haploid gametophytes are also determined by a UV sexual system. During the haploid generation, remarkable sexual dimorphism was exhibited in female and male gametophytes, and a high level of phenotypic differentiation was also found in female and male gametes after gametogenesis [16, 17], which contrasted with the limited levels of sexual dimorphism in *Ectocarpus* gametophytes and gametes. Sex chromosome and SDR genes can play key roles in the evolution of phenotypic differences and speciation [18]. Although a large female chromosome has been reported in several kelp species [19], heteromorphic sex chromosomes have not been found in *S. japonica* until now [20, 21]. After screening the *S. japonica* genomic database by SDR genes of *Ectocarpus*, only a small set of homologs was found [22]. In addition, gene movement into and out of the SDR was observed during the long-term evolution of U/V sex chromosomes in brown algae [10]. Thus, the *S. japonica* SDR may have its own specific structural characteristics and regulatory mechanism for sexual dimorphism. In addition to SDR genes, many sex-related genes are scattered throughout the genome, and their sex-biased expression is likely to account for most sexually dimorphic traits [23]. Herein, sex-biased gene expression is by far the most studied among the mechanisms that generate differences between sexes in invertebrates [24–26], vertebrates [27, 28] and plants [29, 30]. However, genome-wide patterns of sex-biased gene expression have not been identified and characterized in the kelp *S. japonica* thus far. Additionally, obvious phenotypic differentiation was demonstrated in gametes in oogamous Laminariales species. The large and nonmotile female gametes (eggs) can release pheromones, whereas the small and motile male gametes (sperm) can swim directly towards pheromone sources by chemotactic responses [31]. Recently, the sex-dependent transcriptional change during gametogenesis in the sugar kelp *Saccharina latissima* was investigated, providing a molecular level understanding of gametogenesis in kelp [32]. However, it is still necessary to deepen the understanding of how these changes might relate to sex differentiation and to sex-specific fitness at the gamete stage in *S. japonica*.

In this study, comparative transcriptome analysis was performed for male and female gametophytes in different developmental stages. Many sex-biased genes were identified, and the relationship between the abundance of sex-biased genes and the level of sex differentiation was discussed. We also detected a set of sex-specific genes, representing good candidates as SDR genes and sex determination-related genes. The results provide an overview of the sex differential genes in the haploid generation to deepen the understanding of the regulation of sex differentiation in the dioicous kelp *S. japonica*.

## Results

### Sex identification and sexual reproduction of gametophytes

**Fig. 1:**
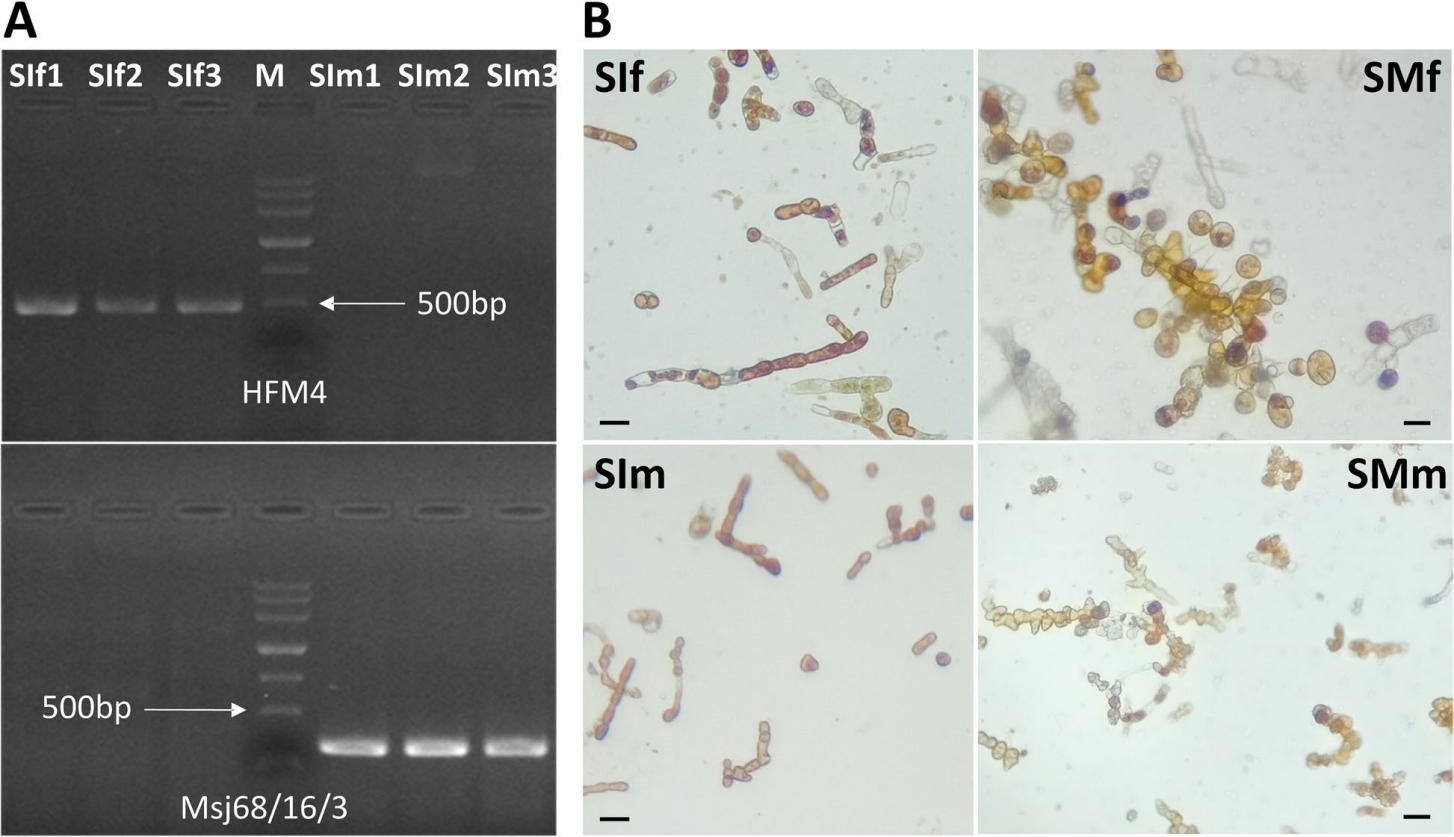
Sex identification of gametophyte samples with sex-specific markers (**A**) and gametophyte development of *Saccharina japonica* (**B**). HFM4, female-specific marker. Msj68/16/3, male-specific marker. SIf, immature female gametophyte on day 0. SIm, immature male gametophyte on day 0. SMf, mature female gametophyte on day 12. SMm, mature male gametophyte on day 12. Bars are 25 μm in (**B**). The agarose gel pictures displayed here are cropped images of the same gel for clearity

Sex-specific markers were used to identify the sex of the samples (Additional file 1: Table S1). The specific PCR products were approximately 500 bp and 300 bp and were amplified only in female (SIf1-SIf3) and male gametophytes (SIm1-SIm3), respectively, showing the reliability of our samples in this study (Fig. 1A). PES medium was used to induce gametophyte development. After 12 days of culture, round eggs on the oogonium in female gametophyte samples (SMf) were clearly observed (Fig. 1B). Antheridia were also visible in male gametophyte samples (SMm).

### Sequencing and mapping of the *S. japonica* transcriptome

Twelve cDNA libraries from male and female gametophytes of *S. japonica* at two different sexual development stages were sequenced, and more than 45 million clean reads were obtained for each sample. Furthermore, 75.88 to 85.87% of the total reads were mapped to the reference genome (Additional file 2: Table S2). Transcript abundances, measured as FPKM, were strongly correlated between biological replicates of each group, with *r* ranging from 0.919 to 0.987 (Additional file 9: Figure S1A). The PCA also showed a good separation of the groups (Additional file 9: Figure S1B).

### Analysis of gene expression during the development of gametophytes

A total of 20,151 genes were expressed (FPKM > 1), of which 13,221 genes were expressed in all sexes and stages (Additional file 9: Figure S1C). At the immature stage, 15,290 and 15,438 genes were expressed in female and male gametophytes, respectively, with a total of 16,218 expressed genes. In contrast, 19,001 genes were expressed at the mature stage. Of them, 16,800 and 17,965 genes were detected in female and male gametophytes, respectively. This indicates that more genes were expressed at the mature stage in *S. japonica* gametophytes.

### Analysis of differentially expressed genes

**Fig. 2:**
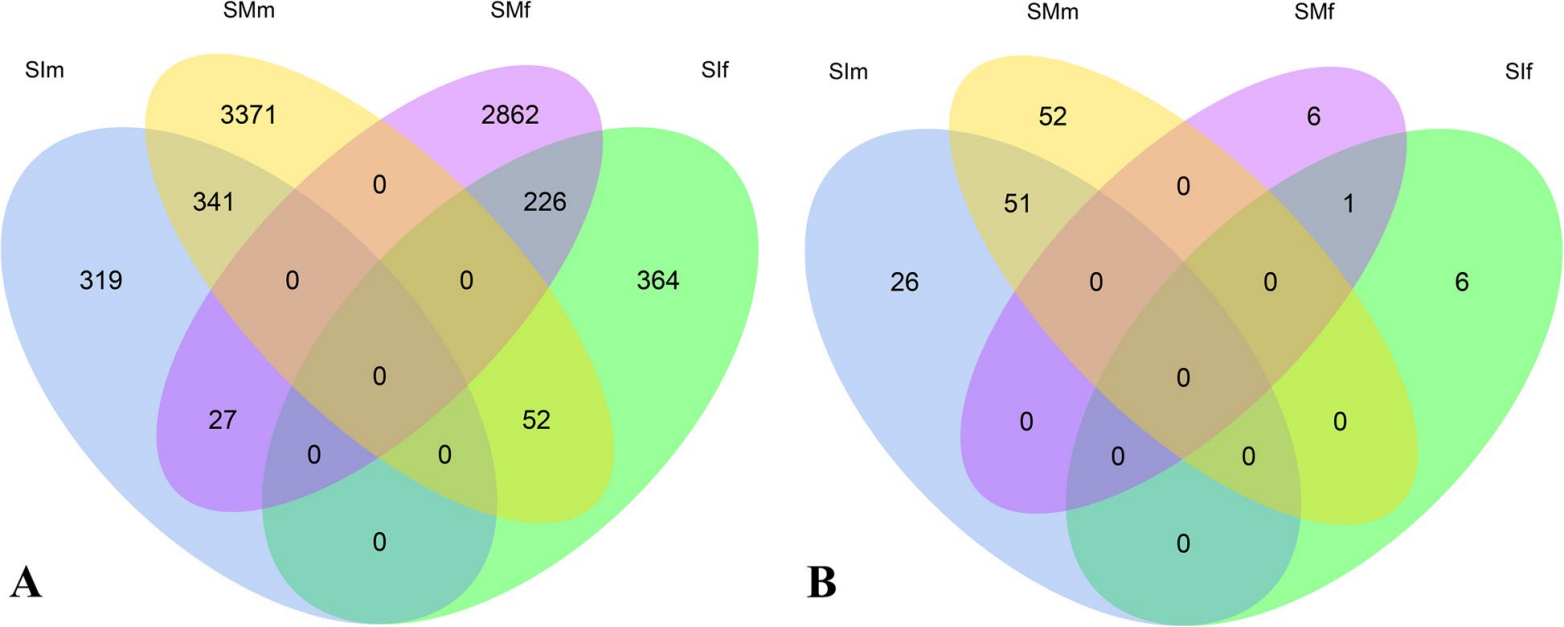
Venn diagram showing overlaps between the sets of sex-biased genes (FPKM > 1, FC > 2, *padj* < 0.05) (**A**) and sex-specific genes (**B**) among the four groups. SIf: immature female gametophytes; SIm: immature male gametophytes; SMf: mature female gametophytes; SMm: mature male gametophytes

**Fig. 3:**
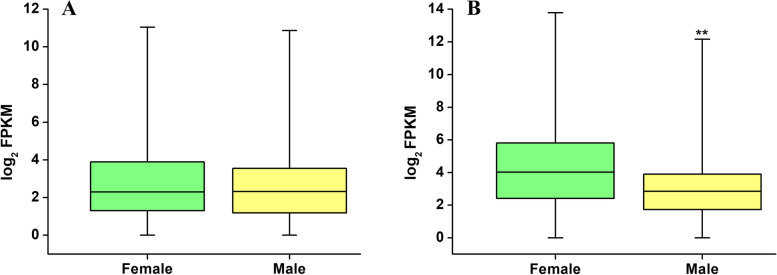
Boxplot showing the mean expression levels (FPKM) of female- and male-biased genes for immature (**A**) and mature (**B**) gametophytes. Asterisks indicate a significant difference (Mann–Whitney U test, *p* < 0.05)

A total of 7,562 DEGs were detected (fold change FC > 2) in pairwise comparisons between the four groups, accounting for 37.53% of all expressed genes identified in this study (Fig. 2A). A heatmap generated using normalized FPKM values and hierarchical clustering revealed that the expression could be classified into several distinct patterns (Additional file 10: Figure S2). In the immature gametophyte stage, only 687 male-biased genes (upregulated DEGs in the SIm group) and 642 female-biased genes (upregulated DEGs in the SIf group) were detected. In contrast, 3,764 male-biased genes (SMm) and 3,115 female-biased genes (SMf) were identified in the mature stage, accounting for 20.95 and 18.54% of all expressed genes, respectively (Table 1; Fig. 2A; Additional file 3: Table S3). The number of sex-biased genes was always higher in the mature stage than in the immature stage, even if the FC threshold for defining DEGs was increased (Table 1). This indicates that more sex-biased genes are involved in gametogenesis and gamete formation than in gametophyte formation. In addition, the proportion of male-biased genes was higher than that of female-biased genes in the mature stage (FC > 2: 20.95 vs 18.54%), and this gap became larger as the FC threshold increased (FC > 10: 6.43 vs 2.89%). However, female-biased genes were expressed at significantly higher levels than male-biased genes in the mature stage (Mann–Whitney U test, *p* < 0.05) (Fig. 3).

**Table 1:** DEGs in immature and mature stages of male and female gametophytes

	SIm DEGs^a^		SIf DEGs		SMm DEGs		SMf DEGs	
	No. gene	Ratio^b^	No. gene	Ratio	No. gene	Ratio	No. gene	Ratio
FC > 2	687	4.45%	642	4.20%	3764	20.95%	3115	18.54%
FC > 4	294	1.90%	334	2.18%	2126	11.83%	1042	6.20%
FC > 6	211	1.37%	212	1.39%	1613	8.98%	728	4.33%
FC > 8	167	1.08%	150	0.98%	1350	7.51%	586	3.49%
FC > 10	148	0.96%	106	0.69%	1156	6.43%	485	2.89%
Total expressed gene (FPKM > 1)	15,438		15,290		17,965		16,800	

^a^upregulated DEGs in SIm group

^b^% of expressed gene

Most of the sex-biased genes showed significant sex-biased expression in only one of the two developmental stages analyzed. Only 8.3% (341/4110) of the male-biased genes and 6.4% (226/3531) of the female-biased genes were differentially expressed in both immature and mature gametophytes (Fig. 2A). In addition, 3.93% (27/687) of the male-biased genes in immature gametophytes were female-biased in mature gametophytes, whereas 8.10% (52/642) of the female-biased genes in immature gametophyte were male-biased in mature gametophytes.

### Functional analysis of sex-biased genes

Gene ontology (GO) terms of sex-biased genes were screened and classified into the cellular component (CC), molecular function (MF) and biological process (BP) categories, respectively. In immature gametophytes, significant enrichment of GO categories was found only for female sex-biased genes. However, more significantly enriched GO terms for both female- and male-biased genes were detected in mature gametophytes (Additional files 4 and 5: Table S4 and S5). There were 34 terms enriched for male-biased genes in mature gametophytes. Most of them were associated with cell movement, including “movement of cell or subcellular component”, “dynein complex”, and “microtubule motor activity”, which were the most significant terms in the BP, CC, and MF categories, respectively. For female-biased genes in mature gametophytes, 110 GO terms were enriched, including “oxidation–reduction process”, “membrane part”, and “oxidoreductase activity” in the BP, CC, and MF categories, respectively (Additional file 11: Figure S3).

The KEGG pathway analysis revealed that significant enrichment of KEGG pathways was only detected for sex-biased genes in the mature stage. The pathway “Phosphatidylinositol signaling system” was the only pathway that was significantly enriched in mature male-biased genes (Additional file 6: Table S6). The mature female-biased genes were allocated to 21 enriched pathways, which were mainly involved in metabolism and photosynthetic processes, including “Metabolic pathways”, “Carbon metabolism”, and “Carbon fixation in photosynthetic organisms”.

#### Cell wall carbohydrate synthesis

**Fig. 4:**
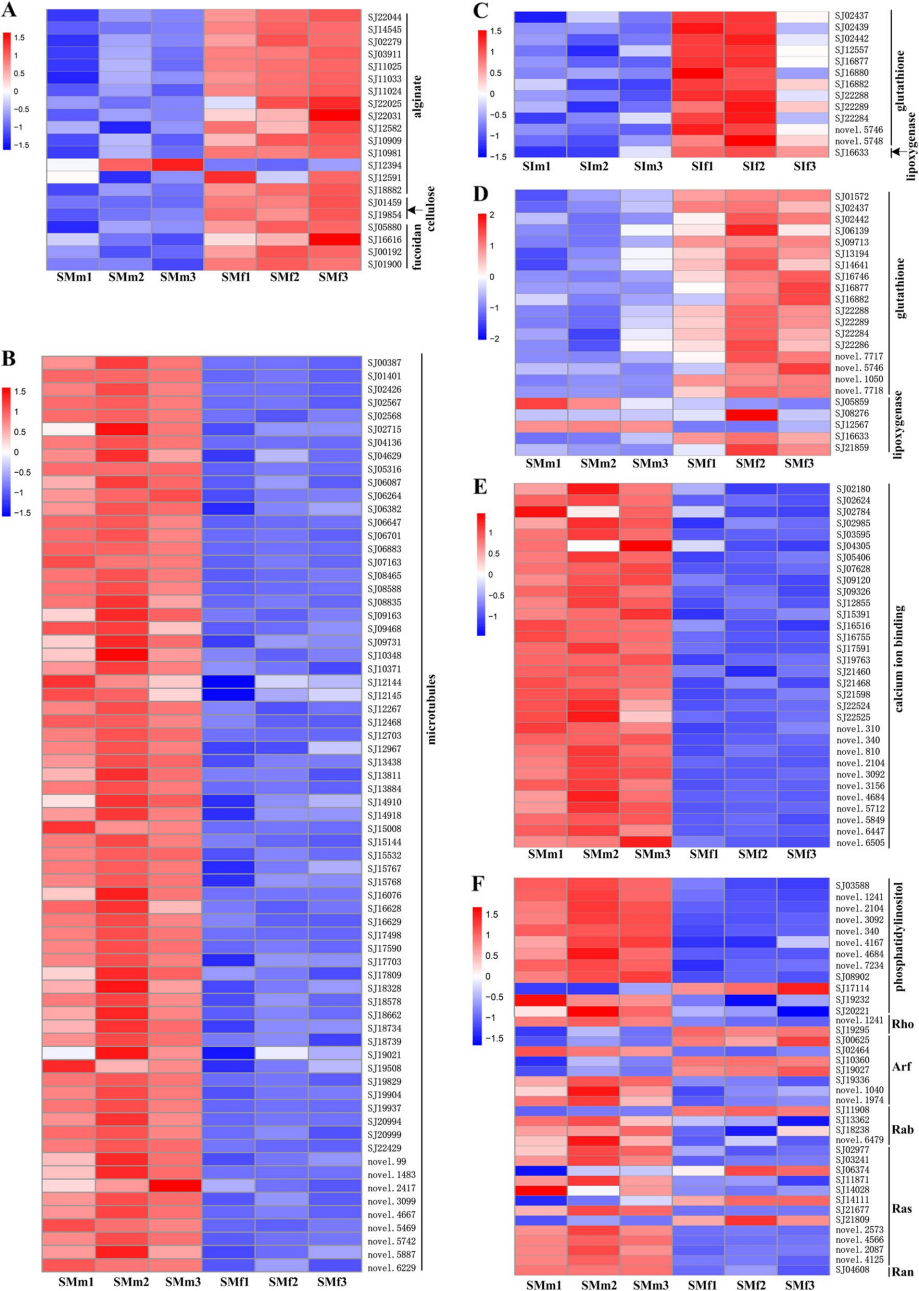
Expression patterns of sex-biased genes in *S. japonica* gametophytes. **A** Cell wall carbohydrates synthesis. **B** Microtubule organization. **C** and **D** Pheromone biosynthesis in immature and mature gametophytes, respectively. **E** Ca^2+^ signal transduction. **F** Phosphatidylinositol signaling pathway. Red indicates upregulated genes, while blue indicates downregulated genes. SIf: immature female gametophytes; SIm: immature male gametophytes; SMf: mature female gametophytes; SMm: mature male gametophytes

Alginate is the most abundant polysaccharide component of the cell wall in brown algae. Most of the sex-biased genes involved in alginate biosynthesis were overexpressed in mature female gametophytes including one mannose-6-phosphate isomerase (MPI) gene *SJ22044*, two phosphomannomutase (PMM) genes *SJ14545* and *SJ02279*, four GDP-mannose 6-dehydrogenase (GMD) genes (*SJ03911*, *SJ11025*, *SJ11033*, and *SJ11024*) and seven mannuronate C5-epimerase (MC5E) genes, whereas one MC5E gene *SJ12394* was downregulated (Fig. 4A). Cellulose and fucoidan are the other polysaccharide components of the cell wall. The expression levels of two cellulose-related genes and four fucoidan-related genes were also higher in mature female gametophytes than in mature male gametophytes (Fig. 4A). These results may be related to primary cell wall biogenesis during the parthenogenesis of female gametophytes. In addition, among the above sex-biased genes at the mature stage, three genes (two MC5E genes, *SJ22025* and *SJ12591*, and one sulfotransferase gene, *SJ01900*) were also female-biased in the immature stage, suggesting that there may be differences in the cell wall polysaccharide composition and content between immature female and mature gametophytes.

#### Microtubule organization

In *S. japonica*, locomotion of sperm depends on the propulsion provided by the flagellum. Correspondingly, many GO terms related to microtubule construction and cell movement were significantly enriched in mature male gametophytes, involving 69 male-biased genes at this stage in this study (Fig. 4B). Of them, only one gene (*novel.2417*) was male-specific, and its expression was extremely low (FPKM < 3). This indicates that flagella-related genes were also preserved in the female gametophyte genome and were expressed when the egg was formed, which is consistent with the expression of flagella-related genes in female *S. latissima* gametophytes [32] and flagellated eggs in *Laminaria angustata* [33]. In addition, two male-biased genes (*SJ17498* and *novel.3099*) in the mature stage were also male-biased in immature male gametophytes. As expected, their expression levels were lower in the immature stage than in the mature stage in both male and female gametophyte. The *SJ17498* gene encodes a protein with a dynein heavy chain domain and may act as a motor for the movement of organelles and vesicles along microtubules. The gene *novel.3099* encodes a protein that contains a myosin and kinesin motor domain, which may be the driving force in myosin- and kinesin-mediated processes.

#### Pheromone biosynthesis

In addition to sperm motility, sperm chemotaxis, which responds to sex pheromones released from eggs, is also crucial for gamete recognition. Brown algal pheromones are C-11 hydrocarbon compounds, and lipoxygenase (LOX) and hydroperoxide lyase (HPL) are involved in their synthesis pathway [34]. In this study, no HPL sex-biased genes were detected, and 3 out of 5 mature sex-biased LOX genes were overexpressed in female gametophytes (Fig. 4D). Among them, the LOX gene *SJ16633* was also upregulated in immature female gametophytes (Fig. 4C), and its encoded protein was highly similar to *Esi0424_0006* LOX from *Ectocarpus*, which may have both LOX and HPL activities [12]. In addition, all glutathione-related DEGs were overexpressed in the female gametophytes, including 12 and 18 female-biased genes in the immature and mature stages, respectively (Fig. 4C and D). This result is consistent with the results observed in *Ectocarpus*, supporting the antioxidation effect of glutathione (as a radical scavenger) in female gametophytes during pheromone synthesis [12].

#### Ca^2+^ signal transduction

It has been reported that a Ca^2+^-dependent pathway through Ca^2+^ channels is involved in chemotactic spermatozoa motility in brown algae [35, 36]. After sensing the sex pheromone gradient, an increase in the cell body and flagellar Ca^2+^ concentration was temporarily induced, and the flagellar waveforms of sperm were changed [37]. In this study, mature female and male gametophytes were cultured separately, and all 32 sex-biased genes enriched in the calcium ion binding term (GO: 0005509) were significantly overexpressed in mature male gametophytes (Fig. 4E). These genes all encode EF-hand domain-containing proteins, which can bind calcium to induce a conformational change and then transmit to their target proteins, often catalyzing enzymatic reactions. This result indicates that the genes involved in calcium signal transduction were regulated before the induction of sex pheromones released from eggs.

#### Phosphatidylinositol signaling pathway

The phosphatidylinositol signaling system, which is involved in converting extracellular signals into intracellular signals, is the only significantly enriched pathway in mature male-biased genes (Additional file 6: Table S6). All eight genes related to this pathway with unknown functions were male-biased (Fig. 4F). In addition, we also identified two genes necessary for phosphoinositide-mediated signaling that were overexpressed in mature male gametophytes. One is a phosphatidylinositol 3- and 4-kinase (PI3K/PI4K) gene (*SJ19232*), which can phosphorylate phosphatidylinositol (PtdIns) on its inositol ring to produce PtdIns3P and PtdIns4P. The other is a phosphoinositide phospholipase C (PI-PLC) gene (*SJ08902*), which cleaves phosphatidylinositol-(4,5)-bisphosphate (PIP2) into the second messenger diacylglycerol and inositol-1,4,5-trisphosphate (IP3) [38, 39]. However, a phosphatidylinositol-4-phosphate 5-kinase (PIP5K) gene (*SJ17114*) involved in the synthesis of PIP2 was downregulated in mature male gametophytes, which is inconsistent with the result observed in *E. siliculosus* [12]. There were also many sex-biased Ras GTPase superfamily genes (Ras, Ran, Rho and Arf) at the mature stage (Fig. 4F). The Rho family GTPases, small G proteins, act as molecular switches shuttling between active and inactive forms and are regulated by two classes of regulatory proteins [40, 41]. As observed in *E. siliculosus* [12], a negative regulator GTP-activating protein (RhoRAP) gene (*novel.1241*) was highly expressed in mature male gametophytes, whereas a positive regulator guanine nucleotide exchange factor (RhoGEF) gene (*SJ19295*) was upregulated in mature female gametophytes in *S. japonica*.

### Comparison of sex-biased genes between *S. japonica* and *E*. sp.

The number of sex-biased genes in *S. japonica* increased greatly in the mature stage. We compared the sex-biased genes in *S. japonica* with those in *E.* sp. with a maximal E value of 1e^−5^. Most of the sex-biased genes were not shared between *S. japonica* and *E.* sp (new sex-biased genes in this study) (Additional file 12: Figure S4). There were 2,408 new female-biased and 3,115 new male-biased genes in *S. japonica*, and 139 new female-biased and 237 new male-biased genes in *E.* sp. Among the homologous genes, some were sex-biased expressed in one of the species (specific sex-biased genes) whereas some were sex-biased expressed in both species (shared sex-biased genes). The proportion of specific sex-biased genes in *S. japonica* (22.0 and 15.5% for female and male, respectively) was higher than that in *E.* sp. (8.0 and 9.3% for female and male, respectively). The proportion of shared sex-biased genes in *S. japonica* (0.7 and 1.6% for female and male, respectively) was lower than that in *E.* sp. (6.7 and 14.7% for female and male, respectively).

### Analysis of sex-specific genes

Among the sex-biased genes, 77 and 103 male specific genes were detected in the immature and mature gametophytes, respectively (Fig. 2B; Additional files 3 and 7: Table S3 and S7). In total, 51 genes were male specific in both the immature and mature gametophyte stages, including a high mobility group-domain encoding gene (*SJ05808*), a membrane transport protein gene (*SJ00352*), a ste20-like kinase gene (*SJ00948*), a plant transposon protein gene (*SJ15019*), an ankyrin repeats protein gene (*SJ13170*), an initiation factor 2 subunit family protein gene (*novel.5218*), and three aconitase family genes (*SJ09771*, *SJ10505* and *novel.2780*). However, there were only 7 female specific genes with low expression at both the immature and mature stages (FPKM < 6) and most of them were newly predicted in this study (Additional files 3 and 7: Table S3 and S7). In total, only one gene with an unknown function (*novel.7290*, partial sequence) was female specific in both the immature and mature gametophyte stages. This is consistent with the previous result that the reference genome is actually a male genome [10] instead of a female genome, as initially reported [42].

### Verification of DEG expression by qRT-PCR

**Fig. 5:**
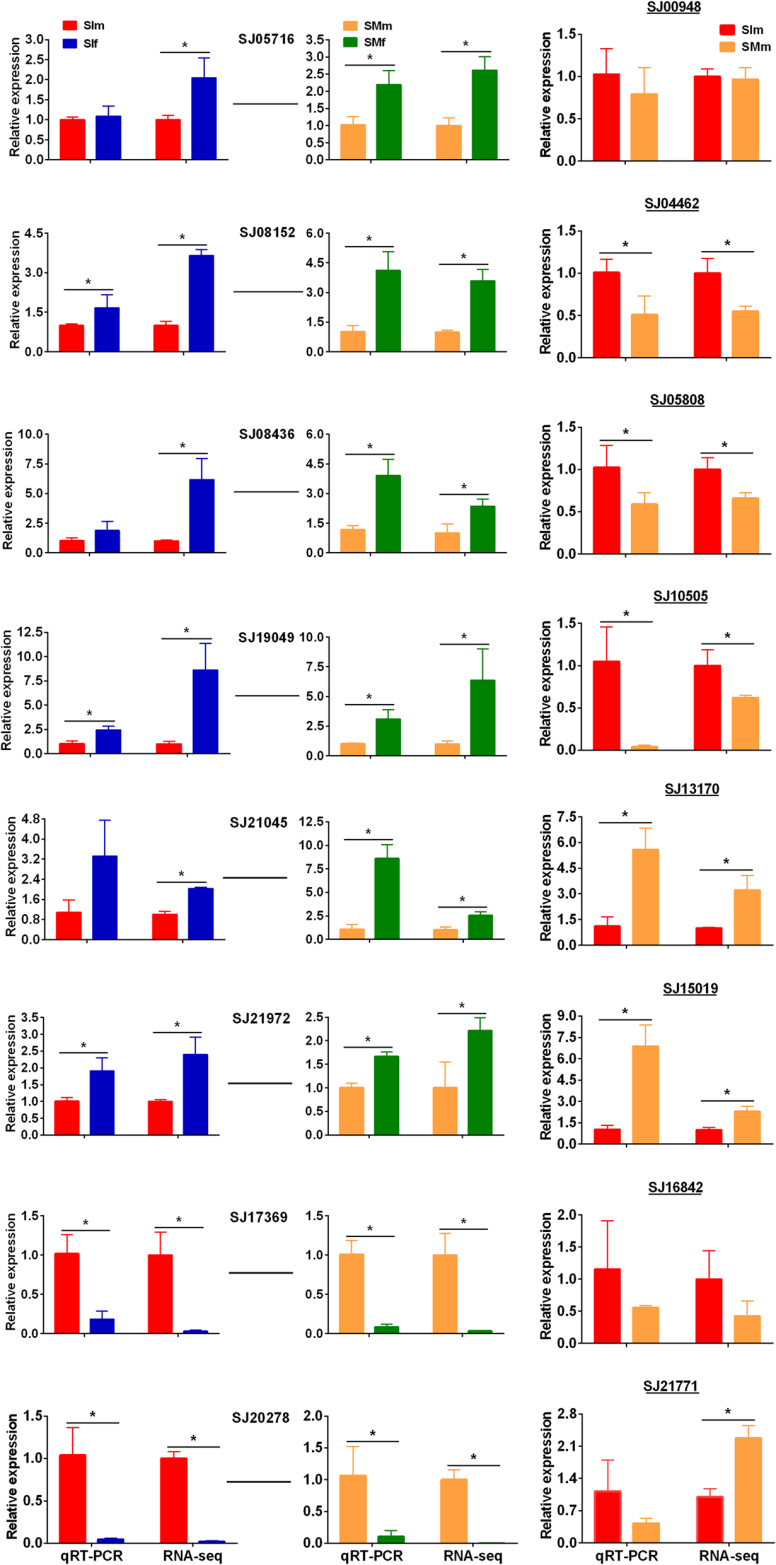
Validation of gene expression by qRT-PCR. Bold genes represent sex-biased genes. Bold and underlined genes represent sex-specific genes. (1) Gene ID: SJ05716- glutaredoxin; (2) Gene ID: SJ08152-unknown; (3) Gene ID: SJ08436-unknown; (4) Gene ID: SJ19049-tyrosinase; (5) Gene ID: SJ21045-chlorophyll A-B binding protein; (6) Gene ID: SJ21972-NAD dependent epimerase; (7) Gene ID: SJ17369-unknown; (8) Gene ID: SJ20278- zinc finger C- × 8-C- × 5-C- × 3-H type family protein; (9) Gene ID: SJ00948-kinase domain protein (10) Gene ID: SJ04462-unknown; (11) Gene ID: SJ05808- high mobility group protein; (12) Gene ID: SJ10505-aconitate hydratase; (13) Gene ID: SJ13170-Ankyrin repeats protein; (14) Gene ID: SJ15019-plant transposon protein; (15) Gene ID: SJ16842-unknown; (16) Gene ID: SJ21771-unknown. SIf: immature female gametophytes; SIm: immature male gametophytes; SMf: mature female gametophytes; SMm: mature male gametophytes. Asterisks indicate a significant difference (t-test, *p* < 0.05)

To validate the gene expression levels during sporophyte development, eight sex-biased genes and eight male-specific genes with high expression level in one of the two stages were selected for qRT-PCR analysis. A single peak in the melting curve was detected in all qRT-PCR amplifications, which indicated that all the PCR products were specifically amplified. For sex-biased genes, the qRT-PCR results were significantly correlated with the RNA-seq results at the immature gametophyte stages (correlation coefficient 0.873) and mature gametophyte stages (correlation coefficient 0.780) (Fig. 5). The expression of male-specific genes was confirmed by qRT-PCR (Additional file 13: Figure S5). In addition, the qRT-PCR results were found to be consistent with the RNA-seq results in different stages of male gametophyte development (correlation coefficient 0.745, Fig. 5). There was still a difference between the results of the two methods. For example, the gene *SJ21771* showed higher expression in mature gametophytes than in immature gametophytes based on RNA-seq results but no difference based on the qRT-PCR results. Generally, the relative transcript levels of the genes examined by qRT-PCR were consistent with those measured by RNA-Seq, indicating the reliability and accuracy of the transcriptome analysis results.

## Discussion

*S. japonica* is a dioicous species in which sex is expressed during the haploid gametophyte generation and remarkable sexual dimorphism is shown in microscopic gametophytes and gametes. In this study, sex-biased genes and sex-specific genes were identified by performing transcriptome sequencing on the male and female gametophytes of *S. japonica* at different stages. Among the 20,151 genes expressed in gametophytes, 37.53% showed sex-biased expression. A similar or larger proportion of sex-biased genes was also revealed in other species with significant sexual dimorphism, including arthropods [43, 44], fish [45], birds [28] and mammals [46]. We found that the high abundance of sex-biased genes in *S. japonica* was mainly attributed to the significant increase in sex-biased genes after gametogenesis. The proportion of sex-biased genes increased from 8.19% (1,329/16,218) in the immature gametophyte stage to 36.20% (6879/19001) in the mature gametophyte stage. In contrast to the results in *S. japonica*, in the isogamous brown alga *Ectocarpus*, less than 12% of the sex-biased genes were detected during haploid gametophyte generation, and more sex-biased genes were found in immature gametophytes than in fertile gametophytes; *Ectocarpus* exhibits low-level sexual dimorphism in gametophytes [8]. Sex-biased gene expression variation by developmental stage has been reported in many plants and animals in previous studies [24, 47, 48]. In this study, the phenotypic difference between the male and female gametophytes was only in the cell size in the immature stage. After gametogenesis, greater phenotypic differences were shown in reproductive organs (oogonia and antheridia) and gametes (eggs and sperm) in *S. japonica* through further sex differentiation. In a previous study of another oogamous species, *S. latissima*, more sex-biased genes were also identified after 8 days of gametogenesis induction culture than under vegetable growth conditions [32]. The functions of sex-biased genes were further predicted in this study. For male-biased genes, no GO terms or KEGG pathways were significantly enriched in the immature stage. However, 34 enriched GO terms were detected in the mature stage, most of which were related to cell movement, microtubule assembly and activity, and calcium ion binding. In addition, the only significantly enriched KEGG pathway was the phosphatidylinositol signaling system. The phosphatidylinositol signaling system has been reported to play a key role in converting extracellular signals into intracellular signals. These results are consistent with the sperm mobility phenotype and its chemotaxis physiological function in the mature stage [31]. For female-biased genes, 110 GO terms and 21 KEGG pathways were enriched in the mature stage. Most cell wall polysaccharide-related genes and pheromone biosynthesis-related genes were overexpressed in female gametophytes, which was also consistent with the egg-specific phenotype (forming cell walls during parthenogenesis) and physiological function (releasing pheromones) [31]. The consistency between the functional differences of sex-biased genes and the phenotypic differentiation provides evidence for the hypothesis that variation in sex-biased gene expression during gametophyte development may be related to the level of sex differentiation in brown algae.

We further compared the sex-biased genes after gametogenesis in *S. japonica* with those in *E.* sp. A core set of sex-biased genes in both *S. japonica* and *E.* sp. were identified as having two sequences from different loci with a maximal E value of 1e^−5^ (Additional file 14: Figure S6). Gene duplication of these sex-biased genes may have occurred in *S. japonica*. For example, seven male-biased dynein heavy chain encoded genes (*SJ06892*, *SJ06883*, *SJ06894*, *SJ06893*, *novel.2365*, *novel.7097* and *SJ06649*) were detected in *S. japonica*, whereas only one (*Ec-25_001350*) was found in *E.* sp. All seven genes were upregulated in males only after gametogenesis. The similar expression patterns of these genes suggest that their functional divergence was not obvious, although gene duplication has contributed to acquiring new genes and creating genetic novelty in brown algae [49–51]. Among the sex-biased genes in *S. japonica*, most of them were not aligned with any gene in the *Ectocarpus* genome and vice versa (Additional file 12: Figure S4). This may be related to the incomplete assembly and annotation of the *S. japonica* reference genome used in this study. This result indicates that more new genes associated with sex differentiation evolved in the *S. japonica* lineage than in the *E.* sp. lineage, according to the large number of sex-biased genes in *S. japonica*. In addition, some specific sex-biased genes were identified. In *S. japonica*, no pathway was enriched by specific male-biased genes (Additional file 15: Figure S7). However, only the “phosphatidylinositol signaling system” pathway was significantly enriched in new male biased genes, which was consistent with the result of all male-biased genes. In addition, 18 pathways were enriched for specific female-biased genes, whereas only six pathways were enriched for newly evolved female-biased genes (Additional file 15: Figure S7). These results suggested that specific sex-biased genes and newly evolved sex-biased genes may play different roles in the different sexes in *S. japonica*.

An excess of male-biased genes has been reported in many species with a male heterogametic sex chromosome system [4, 52, 53], which may be because sexual selection is typically stronger in males than in females. In this study, the number of male-biased genes was similar to that of female-biased genes in immature gametophytes, although a male reference genome was used. This result suggested that there may be no heterotypic sex chromosomes in *S. japonica*. However, more male-biased than female-biased genes were observed in mature gametophytes, which is consistent with the results in *S. latissima* [32]. After gametogenesis, *Saccharina* males need to produce a large number of sperm that can rapidly swim towards the eggs, indicating that there may be more male competition than female competition [54]. The excess of male-biased over female-biased genes in this study may reflect the different levels of sexual selection between the sexes in the dioicous kelp with a UV sex chromosome system.

Compared with sex-biased genes, sex-specific genes may play a more critical role in sex determination and sexual dimorphism. Because the reference genome of *S. japonica* was from a male strain that carried the V sex chromosome but not the U sex chromosome, only one gene was found to be specifically expressed in females in both stages. Hereafter, we focus on male-specific genes. Among the 51 male-specific genes, an HMG domain-containing protein gene (*SJ05808*), a ste20-like protein kinase gene (*SJ00948*) and an aconitase family gene (*SJ10505*) were identified (Additional file 7: Table S7), and their specific expression patterns were confirmed (Additional file 12: Figure S4). They are the putative conserved SDR genes in brown algae [10]. The HMG domain gene is the only gene that is limited to the male SDR across many brown algae species. Many studies have shown that the HMG domain gene in *Ectocarpus*, *Ec-13_001750*, is a particularly strong male sex-determination candidate gene [2, 10, 55]. Our previous study on the characteristics and expression analysis of *SJ05808* also confirmed their potential role in sex determination in *S. japonica* [17]. A ste20 protein kinase has been reported to be necessary for haploid cells to respond to sex pheromones in the yeast *Saccharomyces cerevisiae* [56]; this kinase can transduce the signal induced by the binding of pheromones to membrane receptors in the protein kinase cascade. Aconitase plays an important role in the tricarboxylic acid cycle. The linkage of cytosolic aconitase to the Z chromosome of birds was confirmed in a previous study [57]. In addition, downregulation of the aconitase gene during anther development in sterile plants may affect the energy supply during stamen development and be closely related to male sterility in wheat *Triticum aestivum* [58]. These results indicate that ste20 protein kinase homologs may be involved in pheromone signal transduction by phosphorylation and that aconitase homologs may play a key role in male gamete development in brown algae.

Some male-specific genes in *S. japonica* were not conserved during brown algae SDR evolution. An unknown functional gene (*SJ21771*) was present in the SDR in Laminariales, but its homologous gene was located on an autosome in *Ectocarpus*. The gene *SJ00352* was present in the SDR only in *S. japonica* but was on an autosome or in the pseudoautosomal region (PAR) in other Laminariales species [10]. This gene contained a mem-trans superfamily domain, which indicated that it may encode a member of the auxin efflux carrier protein. In previous studies, it has been shown that the PIN auxin efflux transporter is expressed preferentially in antheridia cells of the green alga *Chara vulgaris* [59] and in male flowers during archespore formation in *Populus tomentose* [60]. These results indicate that *SJ00352* may be involved in sex differentiation and male reproductive development in *S. japonica*.

Six genes (*SJ09422*, *SJ15797*, *SJ15874*, *SJ05597*, *SJ18945* and *SJ13722*) have been reported to translocated out of the SDR in *S. japonica* with an RNA-based retrotransposition mechanism [10]. Corresponding to clear signs of SDR degeneration in the brown algae UV system [6], SDR-to-autosome transposition may be a potential mechanism to counter SDR gene loss [10, 61]. We examined the expression patterns of these genes. They were not differentially expressed (|log_2_ (fold change)|> 1) between males and females, except for the male-biased gene *SJ05597* at the mature stage. In addition, these genes showed diverse expression patterns. We noticed that the *SJ13722* was slightly upregulated in females by 1.64-fold and 1.36-fold at the immature and mature stages, respectively. The *SJ13722* was predicted to encode a Memo-like protein. This protein family may be involved in cell motility by releasing extracellular chemotactic signals [62]. This result suggests that *SJ13722* may still play a functional role in the reproductive process in *S. japonica* and provides evidence for the hypothesis that SDR-to-autosome transposition may be related to the maintenance of parental gene function. Additionally, the previously predicted male SDR genes in *S. japonica* [10] were all included in the 51 male-specific genes in both stages in this study. Thus, our study provided a set of genes for screening male SDR genes. Their biological function and sexual regulation mechanism should be further investigated.

## Conclusions

In this study, a comprehensive survey of the expression patterns of male- and female-biased genes was conducted to understand the regulation of sex development and differentiation in the dioicous brown alga *S. japonica*. More sex-biased genes were detected after gametogenesis than in the immature stage, which was consistent with higher level of sexual dimorphism observed in mature gametophytes. Duplications of sex-biased genes were detected in *S. japonica* by a comparison of the sex-biased genes shared by *S. japonica* and *E.* sp. In addition, many new sex-biased genes evolved in the *S. japonica* lineage, which may contribute to the increase in the number of sex-biased genes in this study. Many sex-biased genes may be related to the regulation of gamete morphogenesis and physiological functions, providing the valuable information for further investigations into sex differentiation. The excess of male-biased over female-biased genes may reflect the different levels of sexual selection between the sexes in the dioicous kelp with a UV sex chromosome system. Furthermore, a set of male-specific genes were also identified, which have great potential as candidate male SDR genes. Their functions and roles in sex determination should be elucidated in further studies in *S. japonica*.

## Methods

### Culture conditions and sample collection

Thirty-six female gametophytes and thirty-six male gametophytes of *Saccharina japonica* were provided by the Kelp Species and Elite Variety Center, Yantai, Shandong (Additional file 8: Table S8). All gametophytes were isolated from farmed varieties in China and preserved in seawater supplemented with 12 μmol NaNO_3_ and 7.35 μmol KH_2_PO_4_ at 10 ± 1 °C under a constant irradiance of 15–20 μmol photos m^−2^ s^−1^. To identify the differentially expressed genes between female and male gametophytes, two experimental groups (three biological replicates for each group) were set up, representing the immature female gametophyte group (SIf) and the immature male gametophyte group (SIm). Each replicate consisted of 12 gametophytes of the same sex. The sex of each gametophyte sample was confirmed by both microscopic examination and sex-specific marker identification [22]. Information on sex-specific markers is listed in Additional file 1: Table S1. To facilitate gametogenesis, the gametophytes in each group were broken down into short fragments and placed in PES medium as described previously [17]. After 12 days of culture, the gametophytes attained sexual maturity (production of oogonia or antheridia) and were collected as two other groups, representing the mature female gametophyte group (SMf) and the mature male gametophyte group (SMm) (Additional file 16: Figure S8). All the experimental samples were flash-frozen in liquid nitrogen and stored at -80 °C before RNA extraction.

### Sex identification of *S. japonica* gametophytes

Sex-specific markers were used to identify the sex of *S. japonica* gametophytes. The male gametophyte was identified by a male specific marker (Msj68/16/3) [22]. According to a female-specific sequence (accession number: MF850255) [22], we designed primer pairs using Primer3 [63, 64] and developed a new female-specific marker in this study. PCR was performed, and amplification was carried out as described in a previous study [22]. The PCR products were separated by electrophoresis on 1.0% agarose gels stained with ethidium bromide.

### RNA extraction and sequencing

Total RNA was extracted using the RNeasy Plant Mini Kit (Qiagen, Hilden, Germany) according to the manufacturer’s instructions. RNA purity and RNA concentration were measured using a Nanodrop 1000 spectrophotometer (Thermo Fisher Scientific, MA, USA). RNA integrity was assessed using the RNA Nano 6000 Assay Kit of the Bioanalyzer 2100 system (Agilent Technologies, CA, USA). Twelve sequencing libraries were generated using the NEBNext® UltraTM RNA Library Prep Kit for Illumina® (NEB, MA, USA) following the manufacturer’s instructions and were sequenced on the Illumina HiSeq 4000 platform with 150 bp paired end reads.

### Mapping of reads to the reference genome and predicting novel transcripts

Clean reads were obtained by removing reads containing adapters, reads containing poly-N and low-quality reads and were aligned to the S. *japonica* reference genome (GenBank number: JXRI00000000.1) using Hisat2 v2.0.5 [65]. The reference genome was actually from a male strain, although it was initially reported as a female. The mapped reads of each sample that had no corresponding annotation information in the reference genome were assembled by StringTie v1.3.6 to predict novel transcripts [66]. The annotation information of the new predicted genes was shown in Additional file 17. StringTie uses a network flow algorithm as well as a de novo assembly step to assemble and quantitate full-length transcripts representing multiple splice variants for each gene locus. FeatureCounts v1.5.0-p3 was used to count the read numbers mapped to each gene [67]. Then, the FPKM of each gene was calculated based on the length of the gene and number of reads mapped to this gene. PCA was performed for all samples to evaluate the difference between groups and the duplication of samples within the group.

### Differential expression analysis

Differentially expressed genes (DEGs) between the two groups were analyzed using the DESeq2 R package (1.16.1) [68]. The resulting P-values were adjusted using Benjamini and Hochberg’s approach for controlling the false discovery rate. An absolute log_2_ (fold change) > 1 and adjusted *P*-value < 0.05 were set as the thresholds to identify significant DEGs. Additionally, genes with FPKM values of 0 in the gametophytes of one sex and larger than 1 in those of another sex were defined as sex-specific genes.

### GO and KEGG enrichment analysis of DEGs

Gene Ontology (GO) categories and KEGG (Kyoto Encyclopedia of Genes and Genomes) enrichment analysis of DEGs were analyzed by the clusterProfiler R package. The terms with corrected *P*-values less than 0.05 were considered significantly enriched.

### Real-time quantitative PCR analysis of DEGs

To estimate the validity of the RNA-seq analysis, the expression levels of 16 predicted DEGs were measured by qRT-PCR. RNA extraction was performed as described above. cDNA was synthesized using the PrimeScript RT Reagent Kit with gDNA Eraser (TaKaRa, Dalian, China). The EF1α gene was used as the reference for internal standardization. All primers were designed using Primer3; they are listed in Additional file 1: Table S1 [63, 64]. qRT-PCR was performed on a CFX96 Real-time PCR Detection System (Bio-Rad Laboratories, CA, USA) using SYBR Premix Ex Taq™ (TaKaRa). The PCR conditions were as follows: an initial step at 95 °C for 30 s; followed by 40 cycles of denaturation at 95 °C for 5 s and annealing at 59 °C for 5 s; and a final dissociation curve analysis at 65 °C for 5 s. Melting curve analysis of the amplification products was performed to demonstrate the specificity of the PCR products. The 2^−ΔΔCt^ method was carried out to analyze the comparative mRNA expression levels [69]. All the data are presented as the mean ± SD (standard deviation of the mean). Statistically significant differences between groups were analyzed by t-test (*p* < *0.05*) using SPSS 19.0 software (SPSS Inc., Chicago, IL, USA). The correlation coefficient between the fold changes in the RNA-seq group and qRT-PCR group was determined with SPSS 19.0 software.

## Supplementary Information

**Additional file 1: Table S1.** List of PCR primers used in this study.

**Additional file 2: Table S2.** Read number and mapping ratio based on RNA-seq data from *S. japonica* gametophytes.

**Additional file 3: Table S3.** Number of sex-biased genes and sex-specific genes in immature and mature gametophytes.

**Additional file 4: Table S4.** Enriched GO terms of male-biased genes in mature gametophytes.

**Additional file 5: Table S5.** Enriched GO terms of female-biased genes in both immature and mature gametophytes.

**Additional file 6: Table S6.** Enriched pathways of sex-biased genes in mature gametophytes.

**Additional file 7: Table S7.** Sex-specific genes of *S. japonica* at the different stages.

**Additional file 8: Table S8.** Information on gametophytes used in this study.

**Additional file 9: Figure S1.** Correlation analysis (A), principal component analysis (PCA) (B) and gene expression analysis (C) during the development of gametophytes. A, Heatmap visualization of Pearson correlation coefficients of log_2_ gene expression between samples. B, PCA between samples. X, Y axis represents the contributor of first component and second component, respectively. The samples in one group shows the same color points. C, Comparison of gene expression in immature and mature gametophytes. SIf and SIm represents the immature female and male gametophytes, respectively. SMf and SMm represents the mature female and male gametophytes, respectively.

**Additional file 10: Figure S2.** Hierarchical clustering of differentially expressed genes (DEGs) based on Z-score normalized FPKM values. Each column represents a group, and each row represents a gene. Blue indicates lower expression and red indicates higher expression.

**Additional file 11: Figure S3.** GO classification of differentially expressed genes (DEGs) between mature male gametophytes (A) and female gametophytes (B). X axis represents the GO term. Y axis represents the significance level of GO term.

**Additional file 12: Figure S4.** Abundance of sex-biased genes from different sources in the mature stage in *S. japonica* (A, male; B, female) and in *Ectocarpus* sp. (C, male; D, female).

**Additional file 13: Figure S5.** Validation of the expression of male-specific genes by qRT-PCR. (1) Gene ID: SJ00948-kinase domain protein (2) Gene ID: SJ04462-unknown; (3) Gene ID: SJ05808- high mobility group protein; (4) Gene ID: SJ10505-aconitate hydratase; (5) Gene ID: SJ13170-Ankyrin repeats protein; (6) Gene ID: SJ15019-plant transposon protein; (7) Gene ID: SJ16842-unknown; (8) Gene ID: SJ21771-unknown. SIf: immature female gametophytes; SIm: immature male gametophytes; SMf: mature female gametophytes; SMm: mature male gametophytes.

**Additional file 14: Figure S6.** Gene duplication of sex-biased genes shared with *S. japonica* and *E.* sp.

**Additional file 15: Figure S7.** Enriched pathways of specific sex-biased genes (A, male; C, female) and newly evolved sex-biased genes (B, male; D, female) in *S. japonica* mature gametophytes. Asterisks indicate a significant difference (*padj* < 0.05)

**Additional file 16: Figure S8**. Culture method and strategy in this study.

**Additional file 17.** Annotation information of new predicted genes in this study.

## Data Availability

All raw RNA sequence read data have been deposited in NCBI (SRA) under accession PRJNA656182 [https://www.ncbi.nlm.nih.gov/bioproject/PRJNA656182].

## Abbreviations

SDR: Sex-determining region
PES: Provasoli-enriched natural seawater
PCA: Principal component analysis
qRT-PCR: Quantitative reverse transcription PCR
FC: Foldchange
HMG: High mobility group
DEGs: Differentially expressed genes
GO: Gene ontology
KEGG: Kyoto encyclopedia of genes and genomes
FPKM: Fragments per kilobase per million
PAR: Pseudoautosomal region
CC: Cellular component
MF: Molecular function
BP: Biological process
MC5E: Mannuronate C5-epimerase
MPI: Mannose-6-phosphate isomerase
PMM: Phosphomannomutase
GMD: GDP-mannose 6-dehydrogenase
LOX: Lipoxygenase
HPL: Hydroperoxide lyase
PI3K/PI4K: Phosphatidylinositol 3- and 4-kinase
PtdIns: Phosphatidylinositol
PI-PLC: Phosphoinositide phospholipase C
PIP2: Phosphatidylinositol-(4, 5)-biophosphate
IP3: Inositol-1, 4, 5-trisphosphate
PIP5K: Phosphatidylinositol-4-phosphate 5-kinase
RhoRAP: GTP-activating protein
RhoGEF: Guanine nucleotide exchange factor
