# Supplementary figures and images for: Differential gene expression patterns during gametophyte development provide insights into sex differentiation in the dioicous kelp Saccharina japonica
Source: BMC Plant Biol. 2021 Jul 14;21:335. doi: 10.1186/s12870-021-03117-z (PMC8278619; doi:10.1186/s12870-021-03117-z)

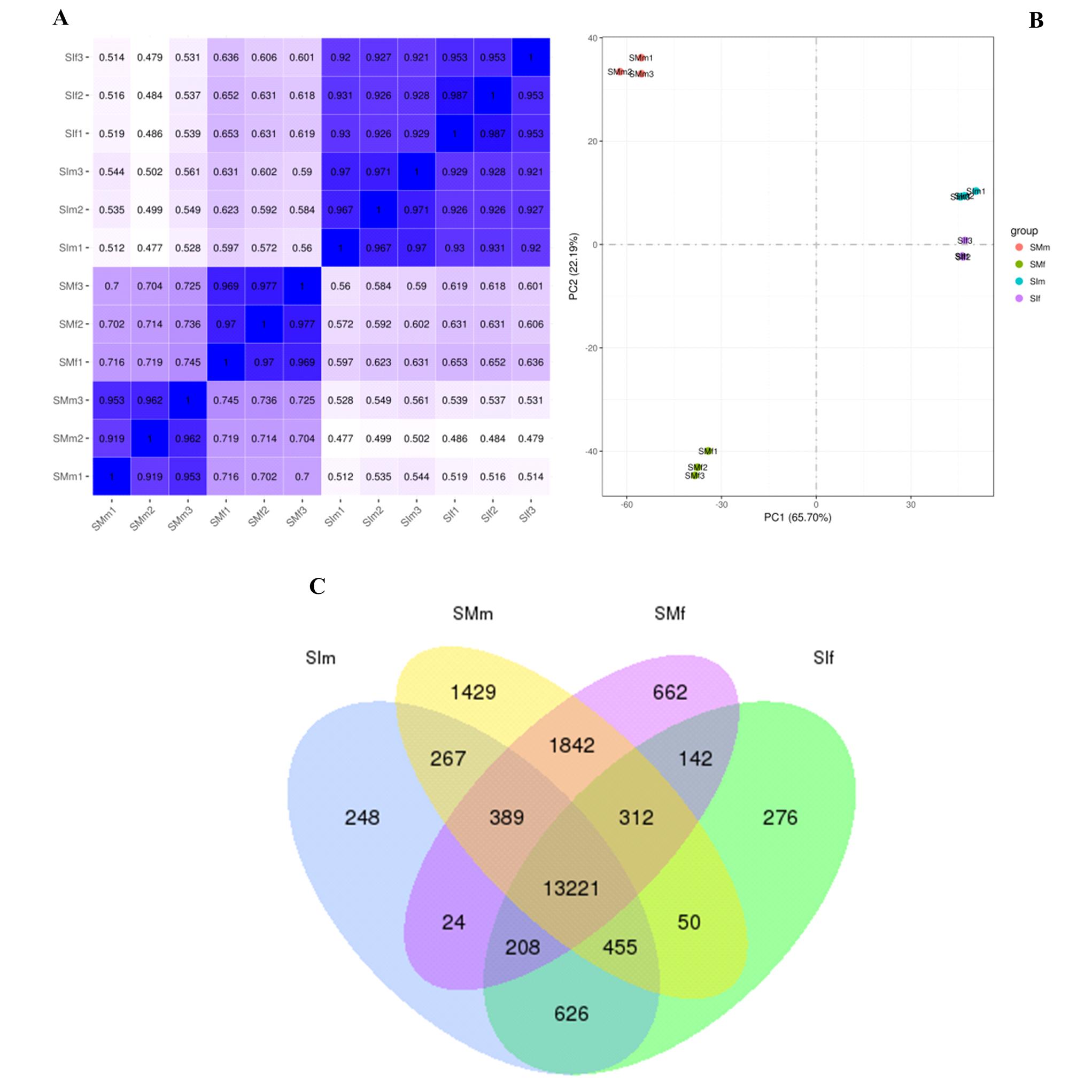

Supplement: Supplementary file 9 — Additional file 9: Figure S1. Correlation analysis (A), principal component analysis (PCA) (B) and gene expression analysis (C) during the development of gametophytes. A, Heatmap visualization of Pearson correlation coefficients of log2 gene expression between samples. B, PCA between samples. X, Y axis represents the contributor of first component and second component, respectively. The samples in one group shows the same color points. C, Comparison of gene expression in immature and mature gametophytes. SIf and SIm represents the immature female and male gametophytes, respectively. SMf and SMm represents the mature female and male gametophytes, respectively. [file 12870_2021_3117_MOESM9_ESM.jpg]

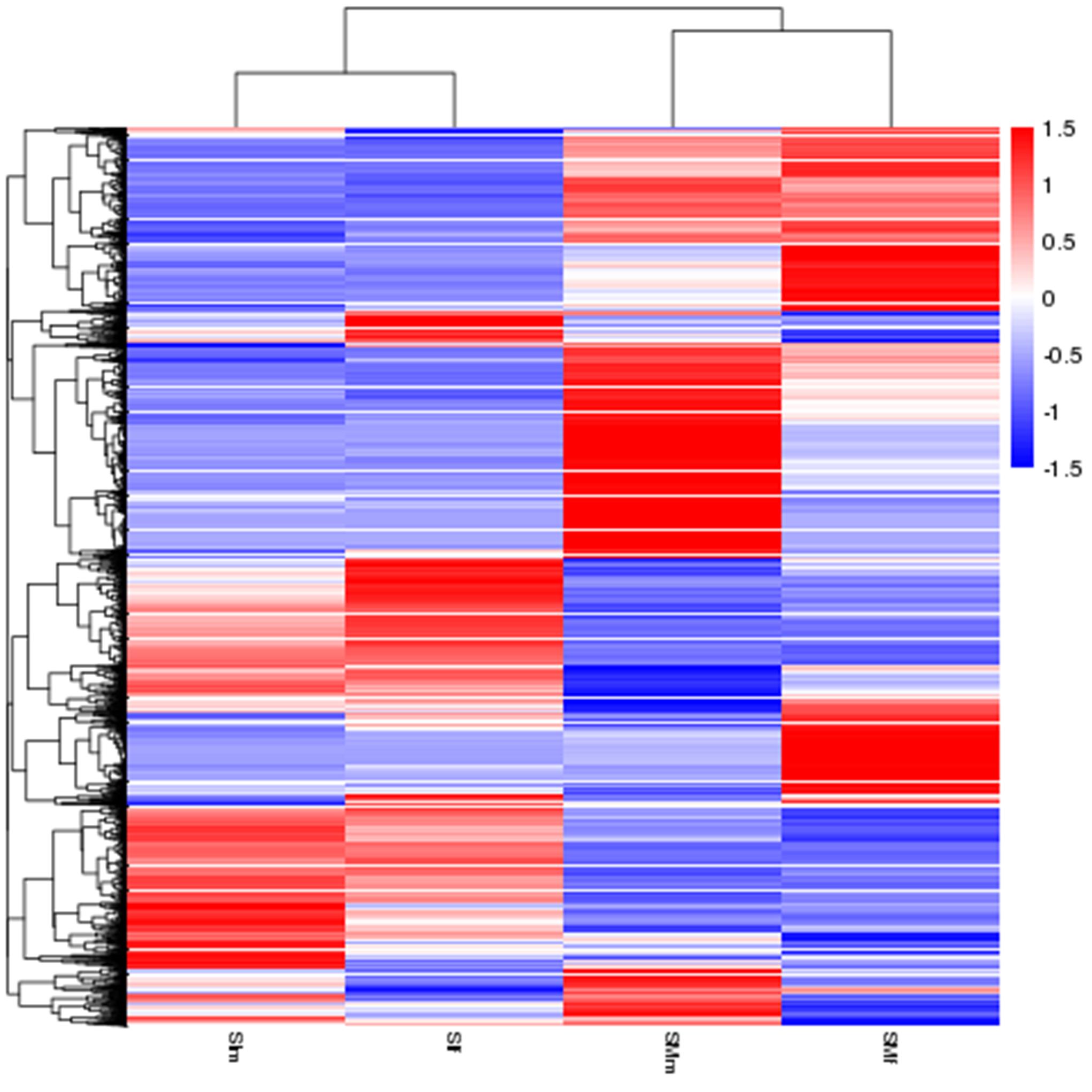

Supplement: Supplementary file 10 — Additional file 10: Figure S2. Hierarchical clustering of differentially expressed genes (DEGs) based on Z-score normalized FPKM values. Each column represents a group, and each row represents a gene. Blue indicates lower expression and red indicates higher expression. [file 12870_2021_3117_MOESM10_ESM.jpg]

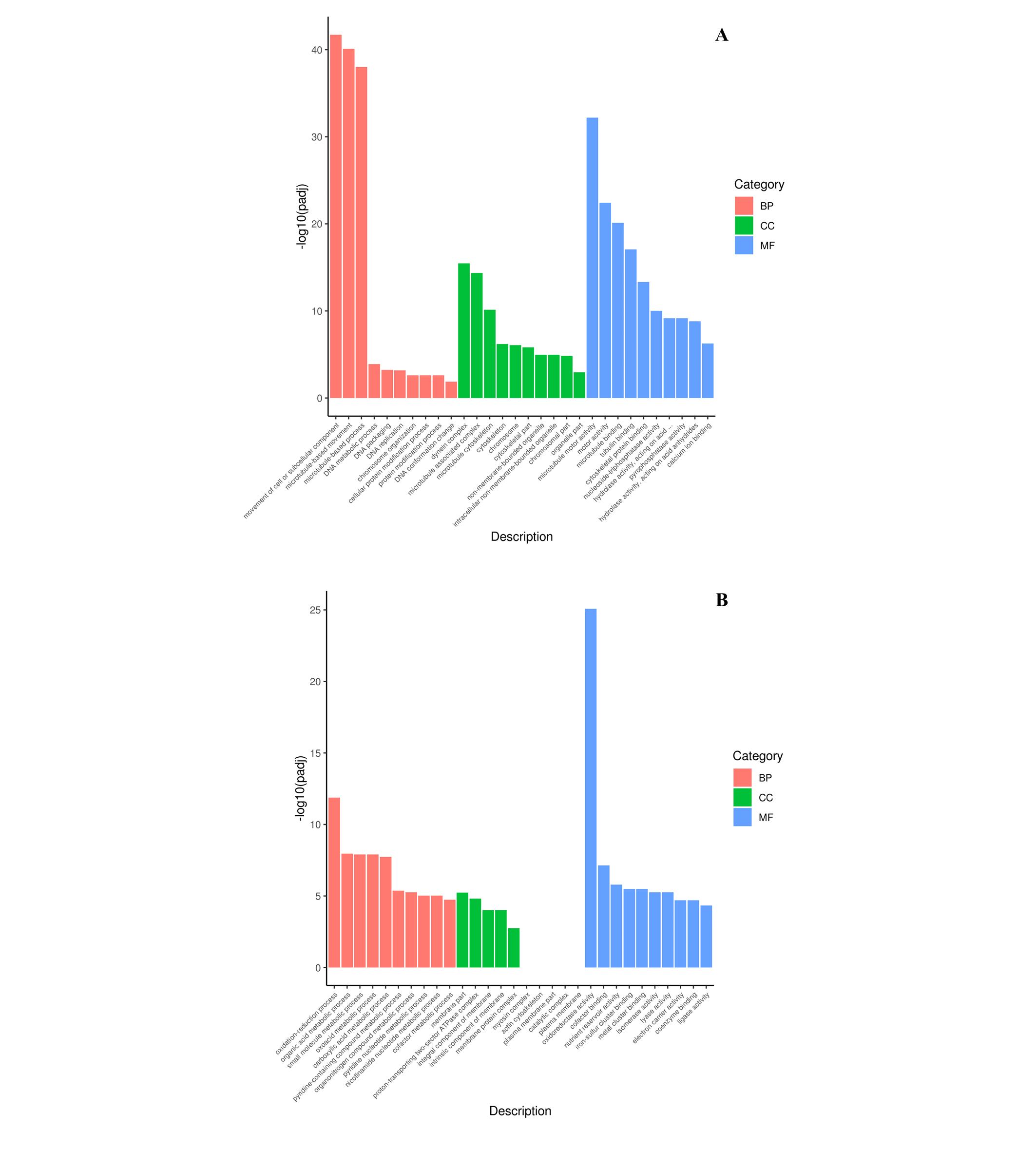

Supplement: Supplementary file 11 — Additional file 11: Figure S3. GO classification of differentially expressed genes (DEGs) between mature male gametophytes (A) and female gametophytes (B). X axis represents the GO term. Y axis represents the significance level of GO term. [file 12870_2021_3117_MOESM11_ESM.jpg]

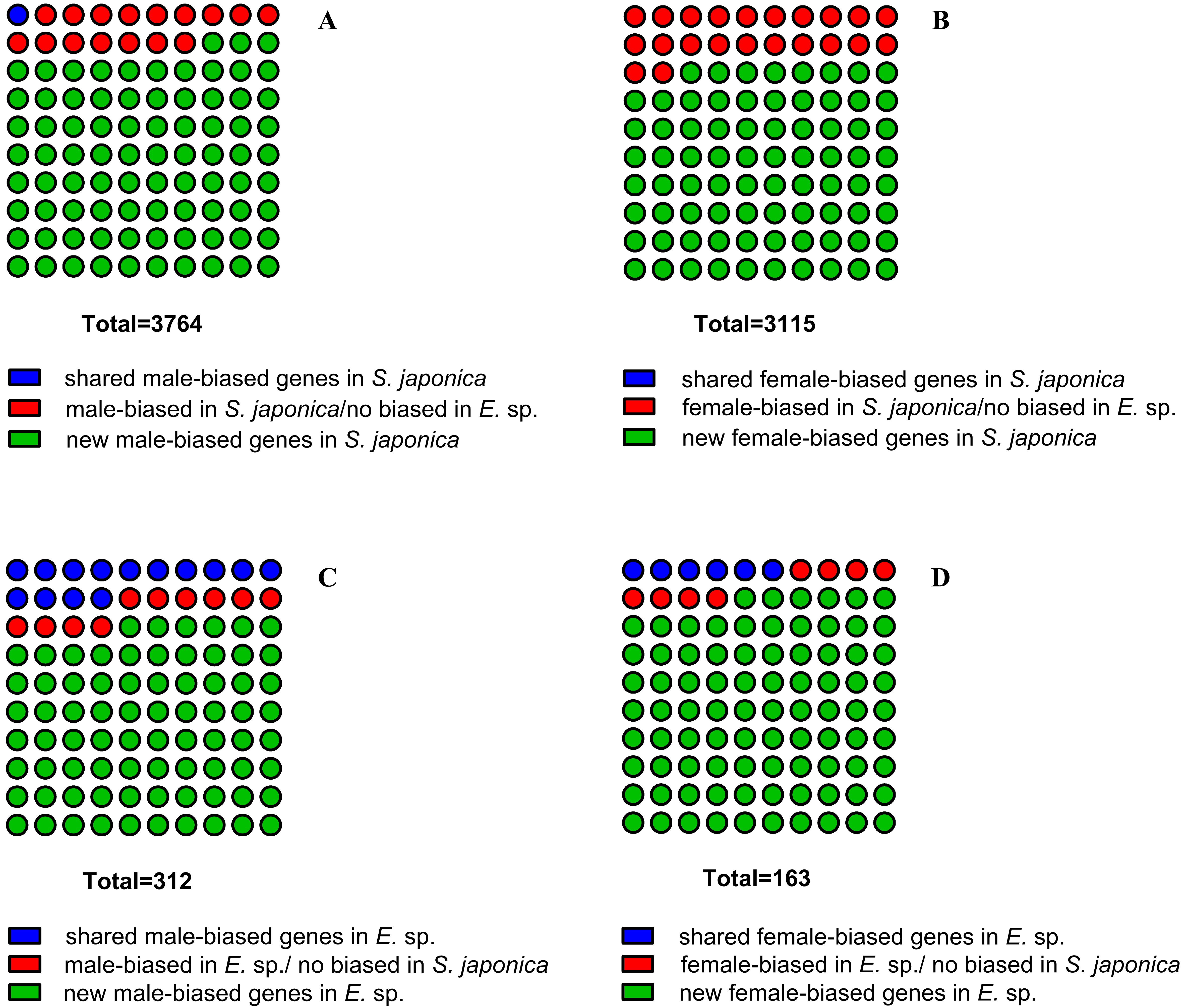

Supplement: Supplementary file 12 — Additional file 12: Figure S4. Abundance of sex-biased genes from different sources in the mature stage in S. japonica (A, male; B, female) and in Ectocarpus sp. (C, male; D, female). [file 12870_2021_3117_MOESM12_ESM.jpg]

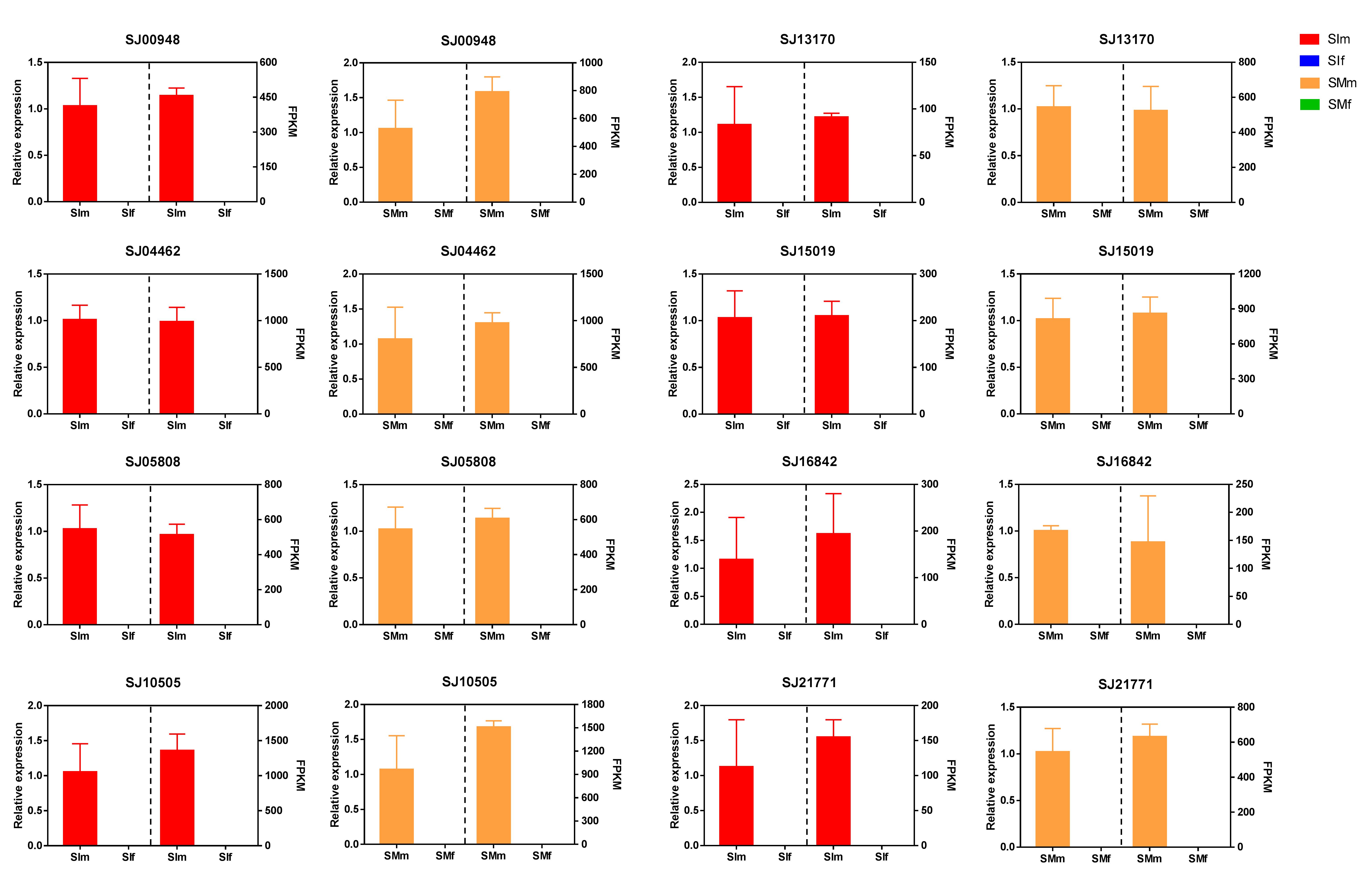

Supplement: Supplementary file 13 — Additional file 13: Figure S5. Validation of the expression of male-specific genes by qRT-PCR. (1) Gene ID: SJ00948-kinase domain protein (2) Gene ID: SJ04462-unknown; (3) Gene ID: SJ05808- high mobility group protein; (4) Gene ID: SJ10505-aconitate hydratase; (5) Gene ID: SJ13170-Ankyrin repeats protein; (6) Gene ID: SJ15019-plant transposon protein; (7) Gene ID: SJ16842-unknown; (8) Gene ID: SJ21771-unknown. SIf: immature female gametophytes; SIm: immature male gametophytes; SMf: mature female gametophytes; SMm: mature male gametophytes. [file 12870_2021_3117_MOESM13_ESM.jpg]

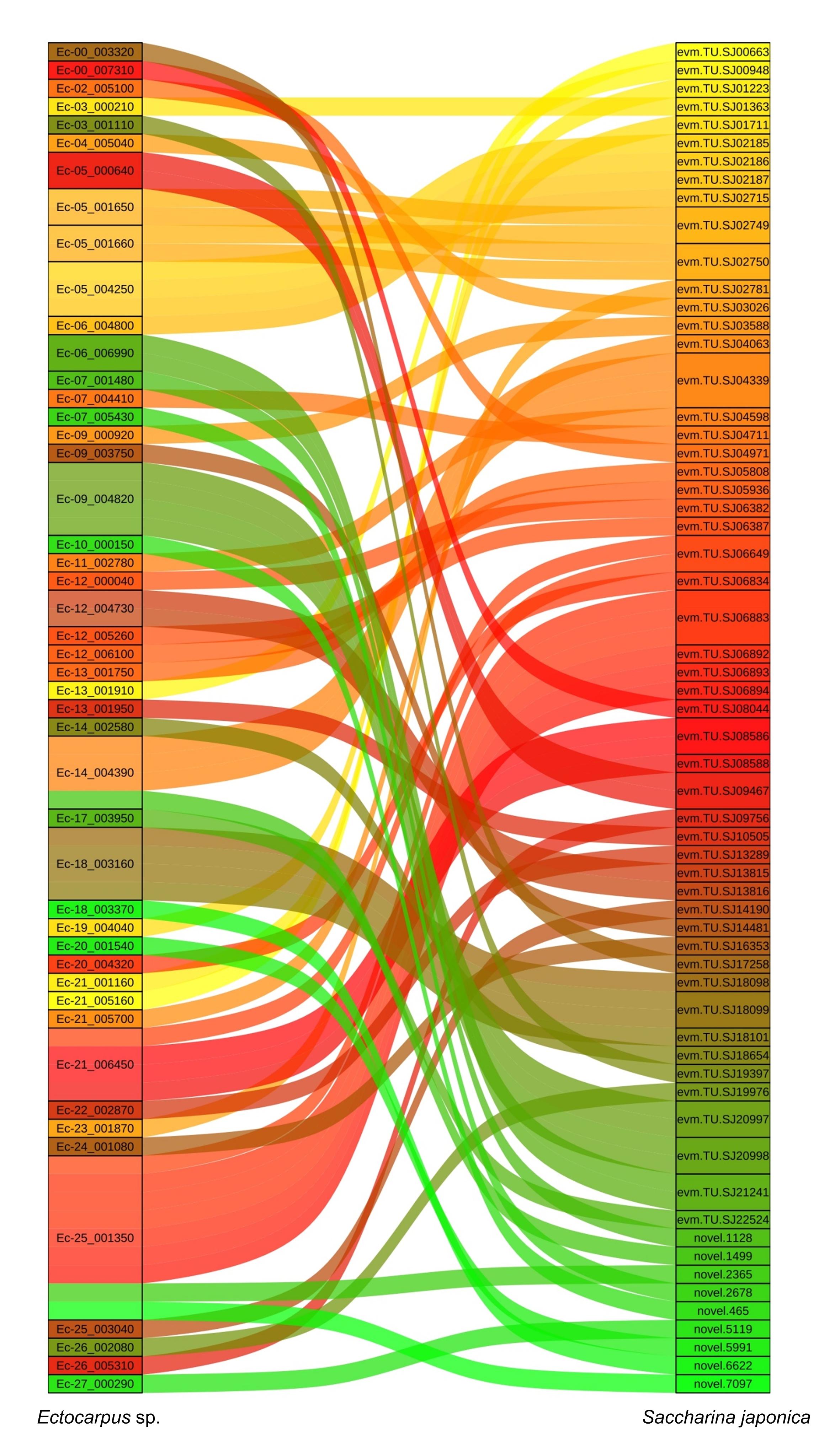

Supplement: Supplementary file 14 — Additional file 14: Figure S6. Gene duplication of sex-biased genes shared with S. japonica and E. sp. [file 12870_2021_3117_MOESM14_ESM.jpg]

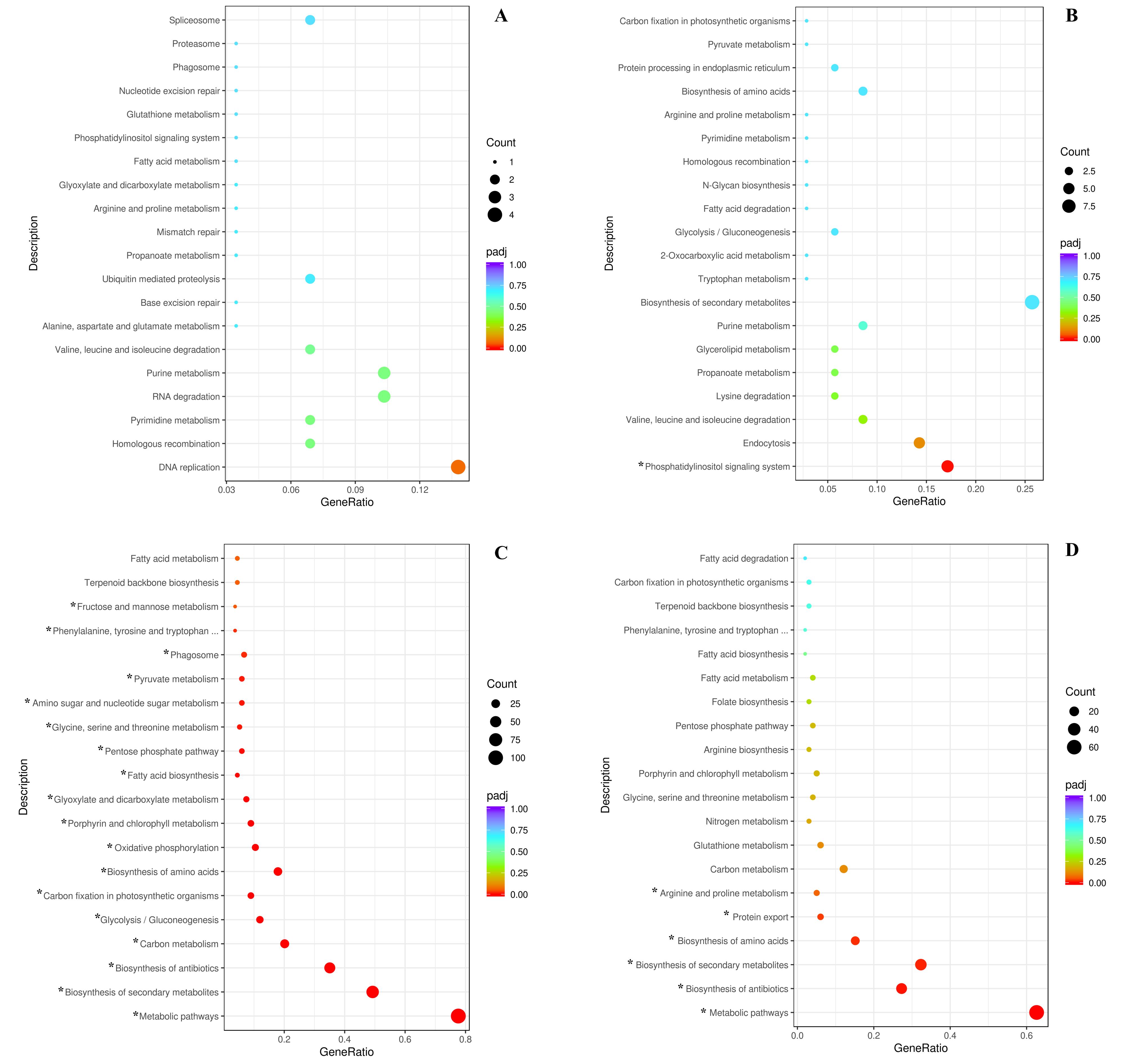

Supplement: Supplementary file 15 — Additional file 15: Figure S7. Enriched pathways of specific sex-biased genes (A, male; C, female) and newly evolved sex-biased genes (B, male; D, female) in S. japonica mature gametophytes. Asterisks indicate a significant difference (padj < 0.05) [file 12870_2021_3117_MOESM15_ESM.jpg]

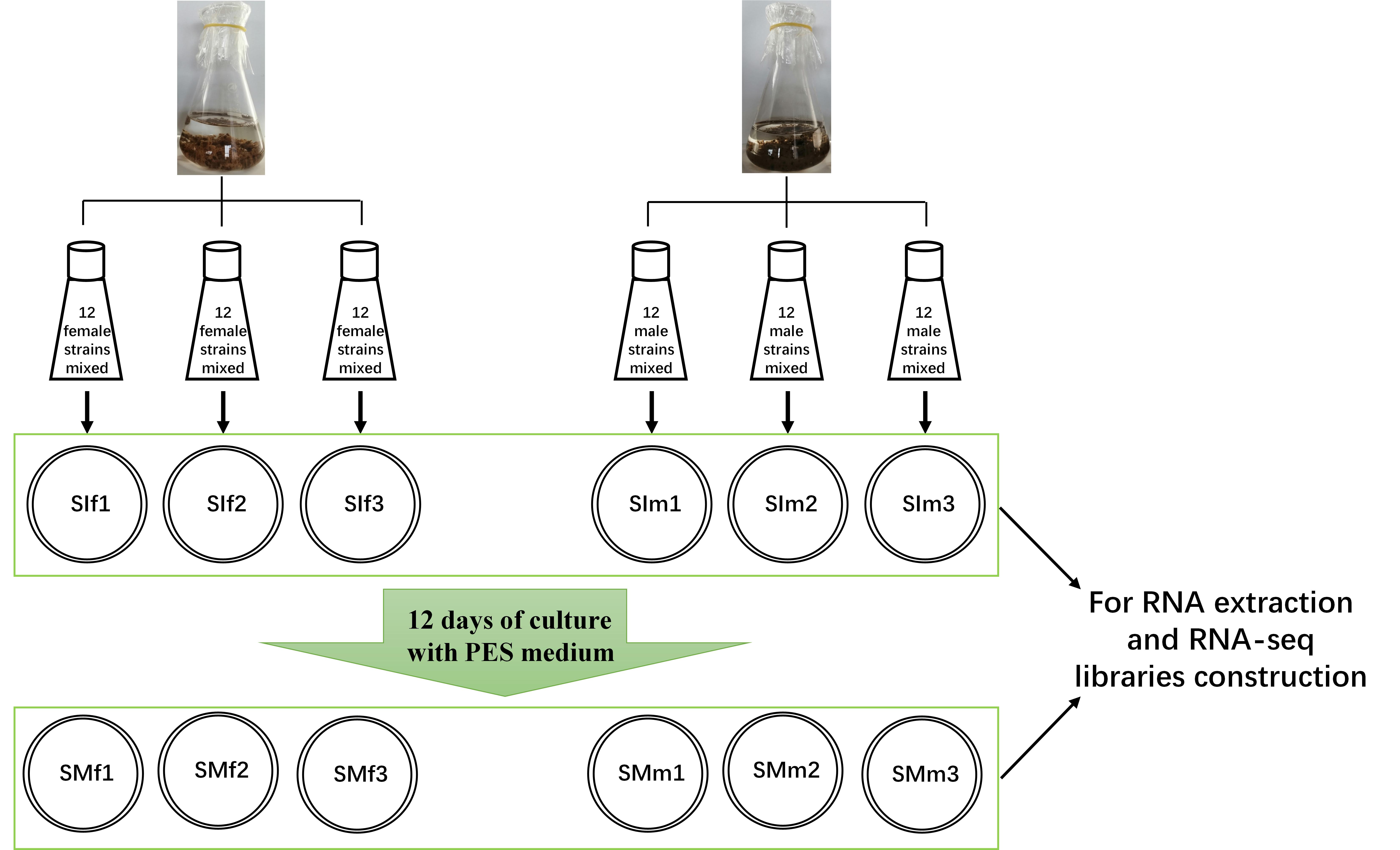

Supplement: Supplementary file 16 — Additional file 16: Figure S8. Culture method and strategy in this study. [file 12870_2021_3117_MOESM16_ESM.jpg]
